# Supplementary figures and images for: Selection, Succession, and Stabilization of Soil Microbial Consortia
Source: mSystems. 2019 May 14;4(4):e00055-19. doi: 10.1128/mSystems.00055-19 (PMC6517688; doi:10.1128/mSystems.00055-19)

**A**

Relative Abundance

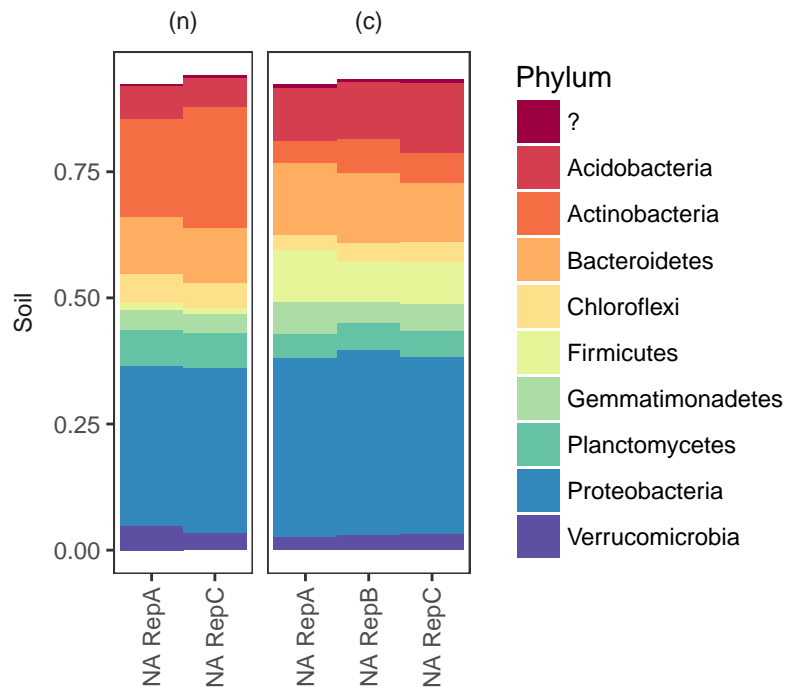**B**

Relative Abundance

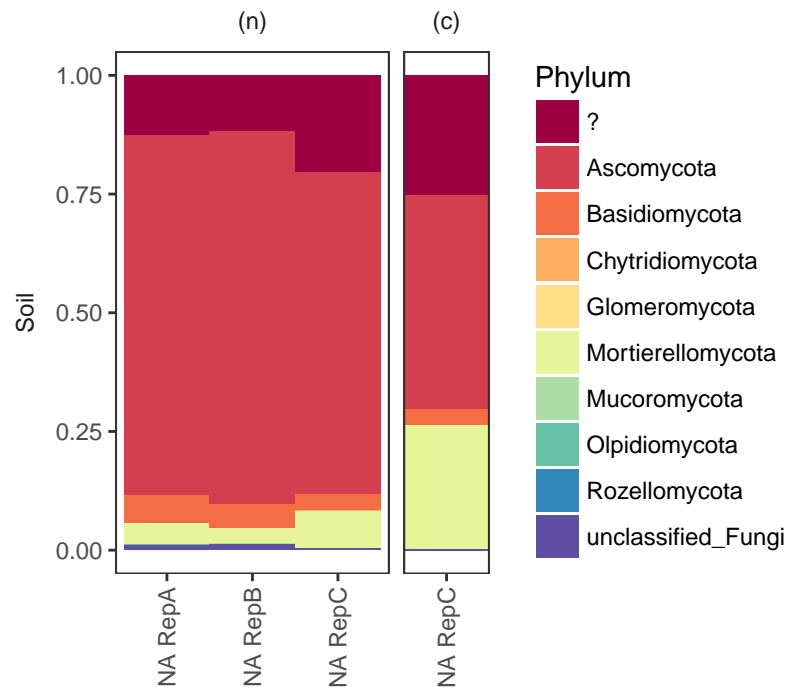**C**

16S OTUs

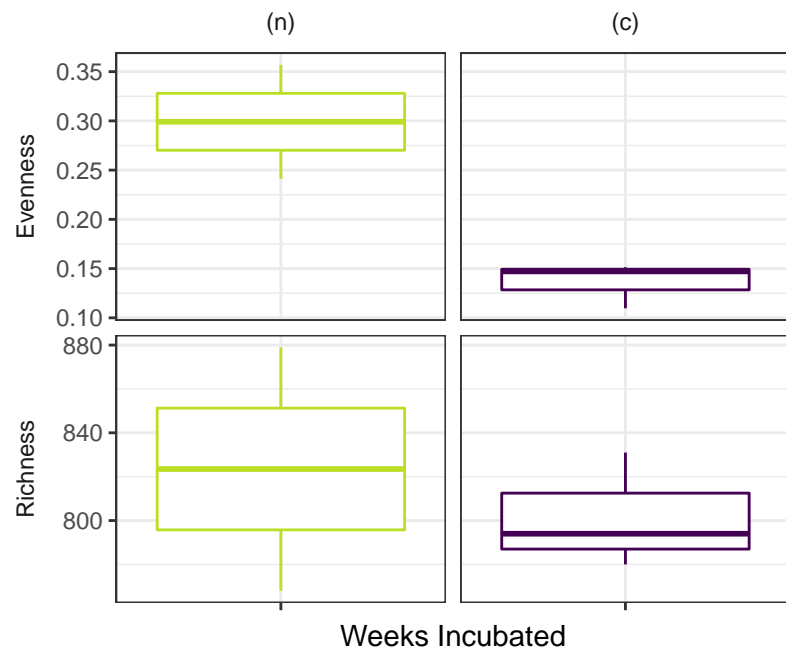**D**

ITS OTUs

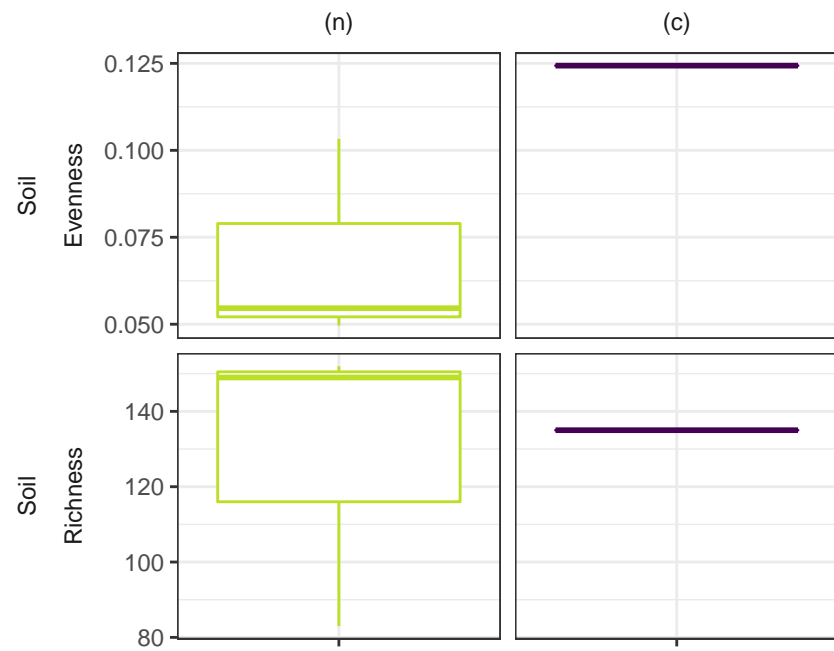

Supplement: FIG S1 [file mSystems.00055-19-sf001.pdf]

# 16S: The most abundant OTUs in negative controls also occur in other samples

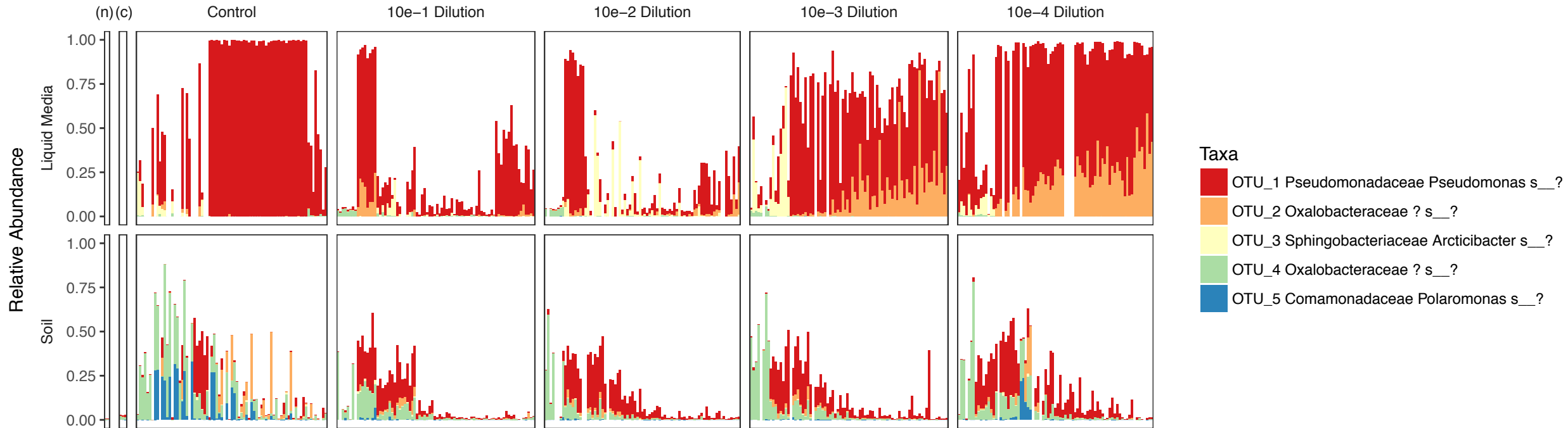

Supplement: FIG S3 [file mSystems.00055-19-sf003.pdf]

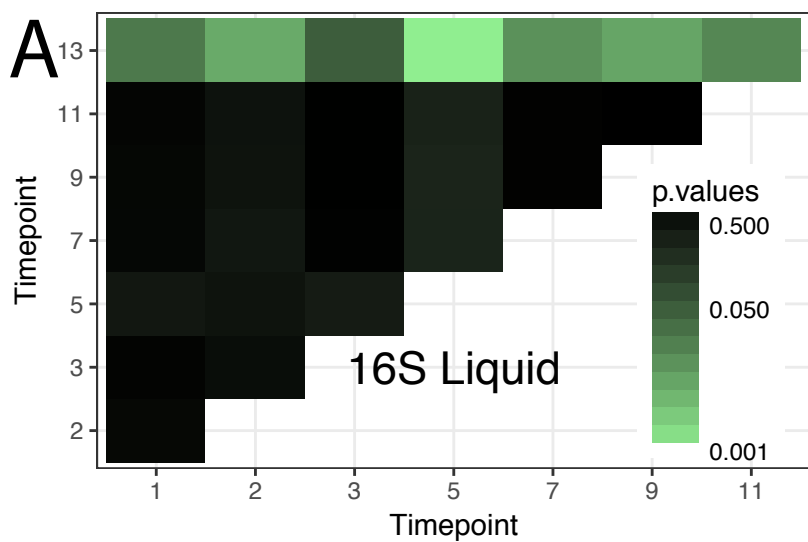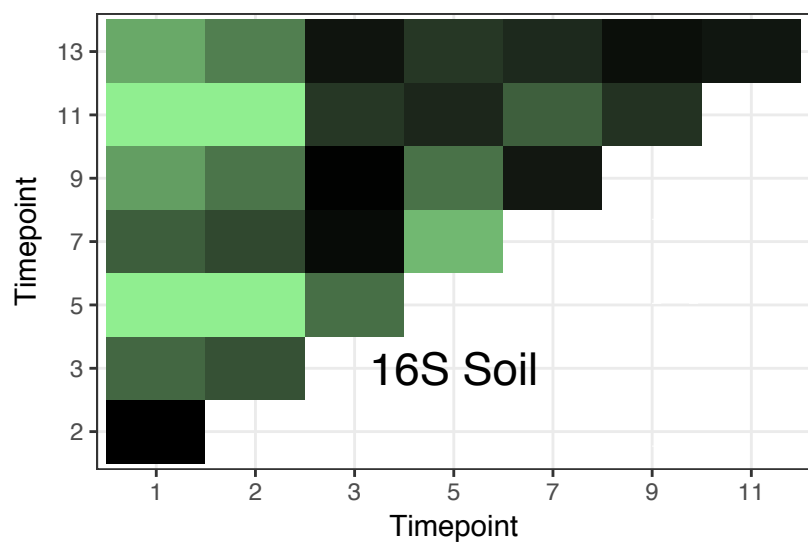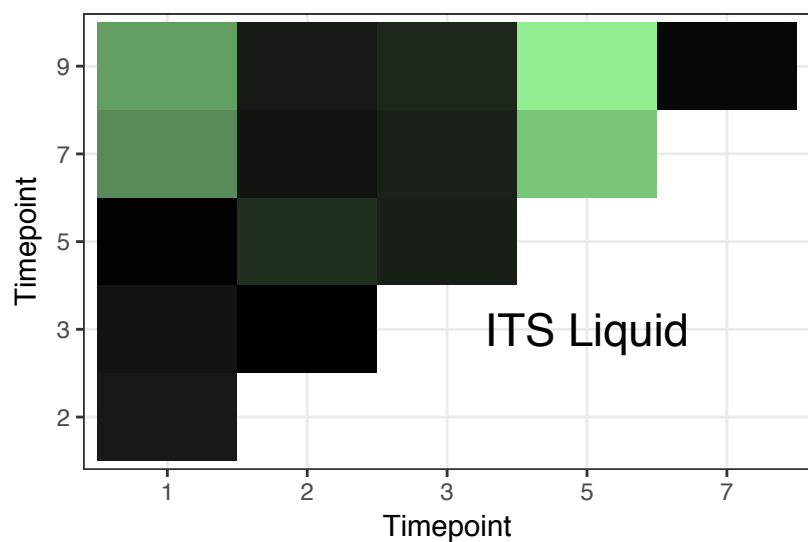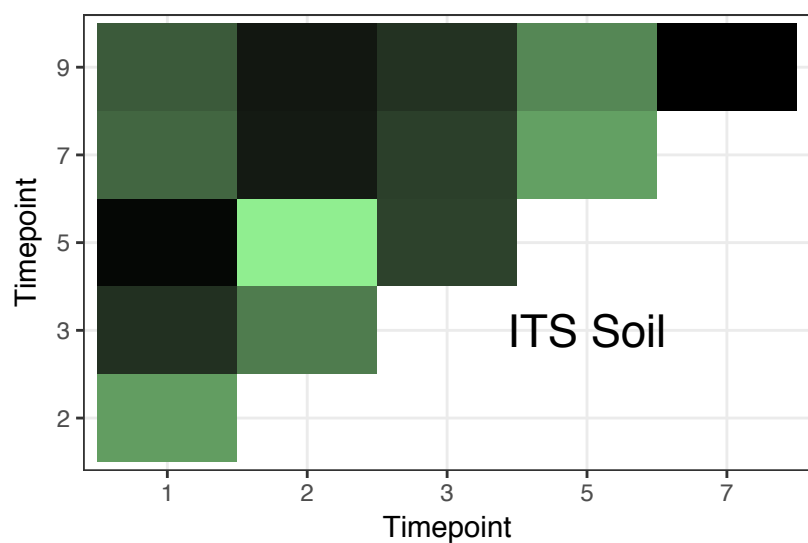

**B** 16S: between replicate variation

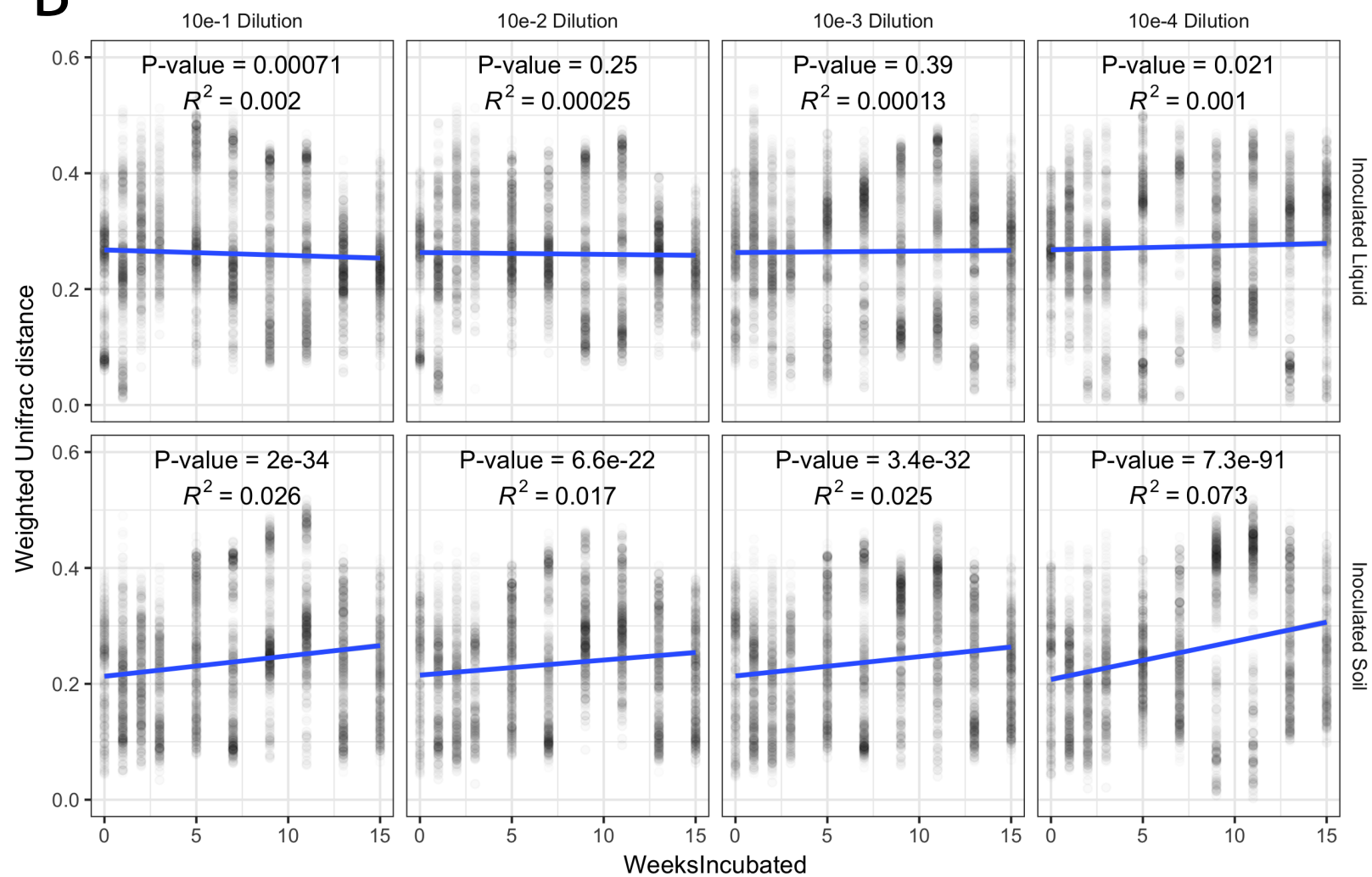

ITS: between replicate variation

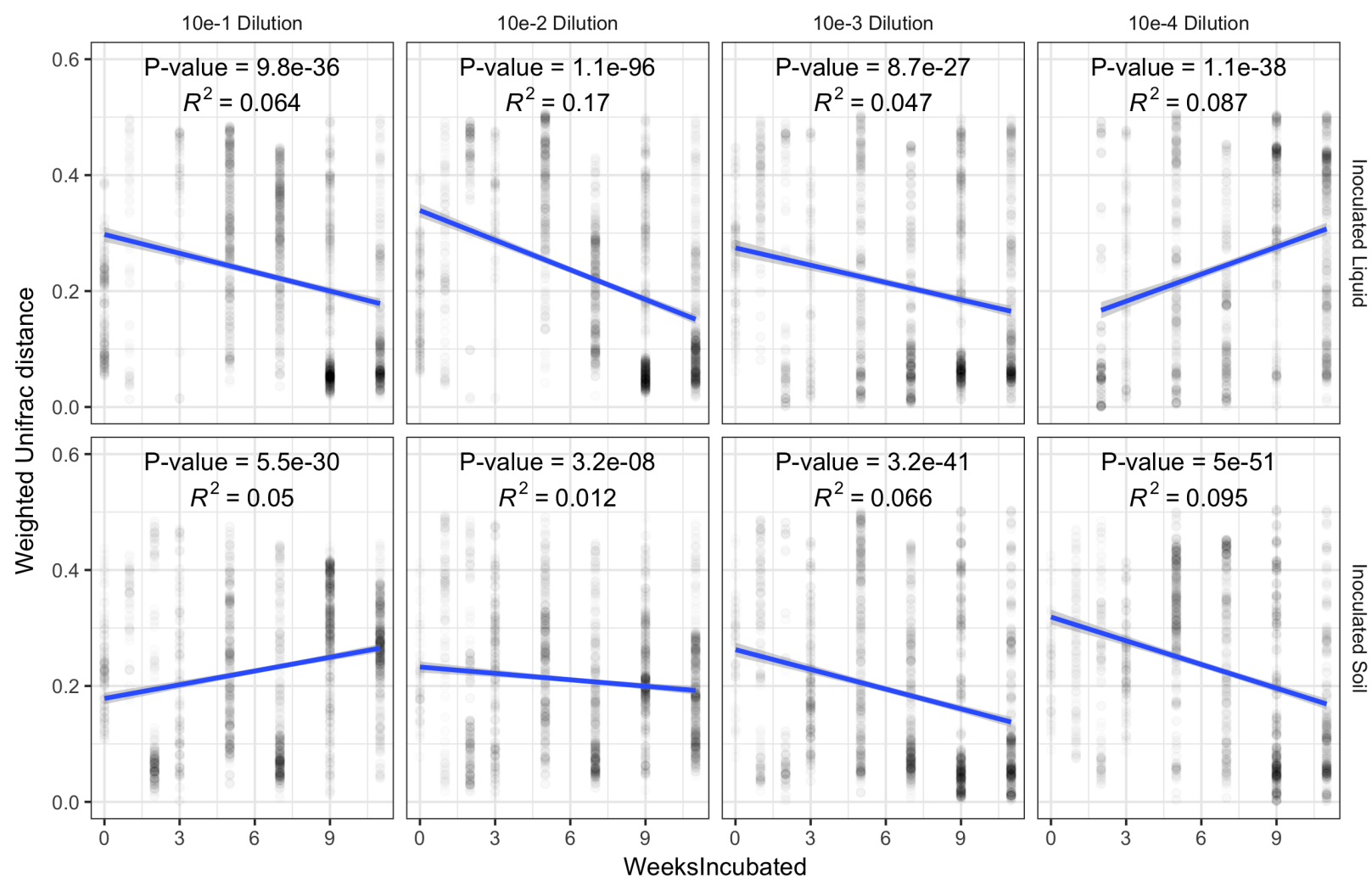

Supplement: FIG S4 [file mSystems.00055-19-sf004.pdf]
