# Supplementary material for: Selection, Succession, and Stabilization of Soil Microbial Consortia
Source: mSystems. 2019 May 14;4(4):e00055-19. doi: 10.1128/mSystems.00055-19 (PMC6517688; doi:10.1128/mSystems.00055-19)
Supplement: FIG S2 [file mSystems.00055-19-sf002.pdf]

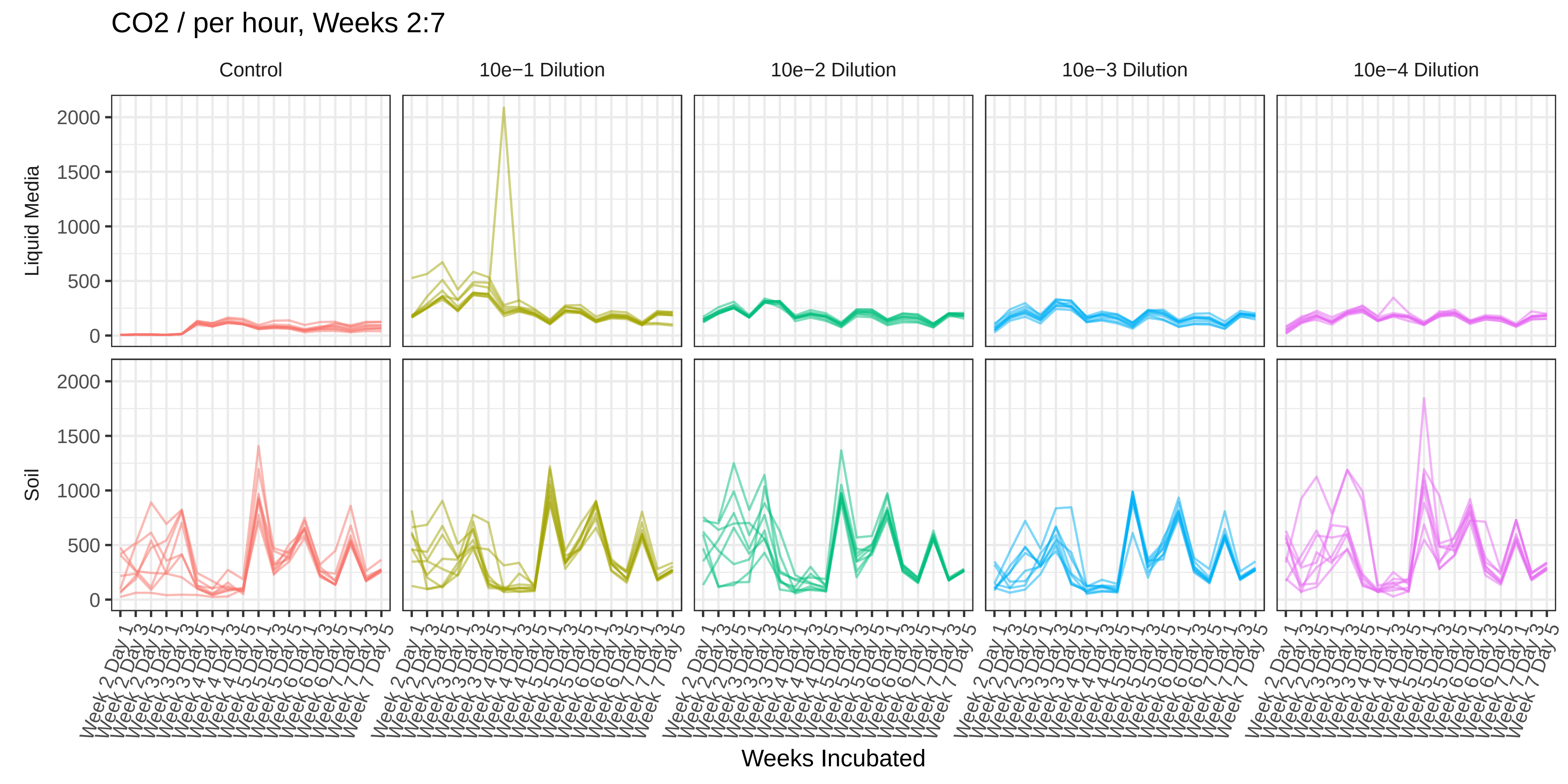
CO2 / per hour, Weeks 2:7
Control
10e−1 Dilution
10e−2 Dilution
10e−3 Dilution
10e−4 Dilution
Liquid Media
Soil
2000
1500
1000
500
0
Week 2 Day 1
Week 2 Day 3
Week 2 Day 5
Week 3 Day 1
Week 3 Day 3
Week 3 Day 5
Week 4 Day 1
Week 4 Day 3
Week 4 Day 5
Week 5 Day 1
Week 5 Day 3
Week 5 Day 5
Week 6 Day 1
Week 6 Day 3
Week 6 Day 5
Week 7 Day 1
Week 7 Day 3
Week 7 Day 5
Weeks Incubated
