# Supplementary material for: Selection, Succession, and Stabilization of Soil Microbial Consortia
Source: mSystems. 2019 May 14;4(4):e00055-19. doi: 10.1128/mSystems.00055-19 (PMC6517688; doi:10.1128/mSystems.00055-19)
Supplement: TABLE S1 [file mSystems.00055-19-st001.docx]

| **Type** | **Treatment** | **10e-2 Dilution - 10e-1 Dilution** | **10e-3 Dilution - 10e-1 Dilution** | **10e-3 Dilution - 10e-2 Dilution** | **10e-4 Dilution - 10e-1 Dilution** | **10e-4 Dilution - 10e-2 Dilution** | **10e-4 Dilution - 10e-3 Dilution** |
| --- | --- | --- | --- | --- | --- | --- | --- |
| 16S | Soil | 0.002598 | 0 | 0 | 0 | 0 | 0.1138814 |
| 16S | Liquid | 0 | 0 | 0 | 0 | 0 | 0.0000024 |
| ITS | Soil | 0.0007323 | 0 | 0.0000002 | 0.0009251 | 0.9998387 | 0.0000002 |
| ITS | Liquid | 0 | 0 | 0 | 0 | 0 | 0.0014198 |
