# Supplementary material for: Selection, Succession, and Stabilization of Soil Microbial Consortia
Source: mSystems. 2019 May 14;4(4):e00055-19. doi: 10.1128/mSystems.00055-19 (PMC6517688; doi:10.1128/mSystems.00055-19)
Supplement: TABLE S2 [file mSystems.00055-19-st002.docx]

|  |  |  |  |  |
| --- | --- | --- | --- | --- |
| **Type** | **Treatment** | **R2** | **p.value** |  |
| 16S | Inoculated Soil | 0.3109907 | 0.001 |  |
| 16S | Inoculated Liquid | 0.7656066 | 0.001 |  |
| ITS | Inoculated Soil | 0.2955797 | 0.008 |  |
| ITS | Inoculated Liquid | 0.6843260 | 0.001 |  |
