# Supplementary material for: Selection, Succession, and Stabilization of Soil Microbial Consortia
Source: mSystems. 2019 May 14;4(4):e00055-19. doi: 10.1128/mSystems.00055-19 (PMC6517688; doi:10.1128/mSystems.00055-19)
Supplement: TABLE S3 [file mSystems.00055-19-st003.docx]

**> dist.t1 # 16S liquid**

Permutation test for homogeneity of multivariate dispersions

Permutation: free

Number of permutations: 999

Response: Distances

Df Sum Sq Mean Sq F N.Perm Pr(>F)

Groups 7 0.10181 0.0145443 1.8706 999 0.076 .

Residuals 230 1.78834 0.0077754

---

Signif. codes: 0 ‘***’ 0.001 ‘**’ 0.01 ‘*’ 0.05 ‘.’ 0.1 ‘ ’ 1

Pairwise comparisons:

(Observed p-value below diagonal, permuted p-value above diagonal)

1 2 3 5 7 9 11 13

1 0.8570000 0.9010000 0.5530000 0.8400000 0.8880000 0.8960000 0.030

2 0.8408961 0.7740000 0.6240000 0.6050000 0.6170000 0.6670000 0.008

3 0.9265345 0.7727334 0.5420000 0.9460000 0.9870000 0.9900000 0.056

5 0.5615476 0.6364887 0.5304231 0.3310000 0.3530000 0.4290000 0.003

7 0.8517255 0.6004488 0.9517810 0.3350217 0.9540000 0.9660000 0.010

9 0.8858396 0.6537449 0.9847483 0.3672397 0.9510812 1.0000000 0.013

11 0.8932968 0.6887733 0.9862912 0.4095452 0.9556976 0.9992465 0.017

13 0.0266473 0.0043442 0.0578710 0.0016131 0.0122193 0.0104359 0.0176215

**> dist.t2 # ITS liquid**

Permutation test for homogeneity of multivariate dispersions

Permutation: free

Number of permutations: 999

Response: Distances

Df Sum Sq Mean Sq F N.Perm Pr(>F)

Groups 5 0.3227 0.064534 1.4987 999 0.211

Residuals 111 4.7796 0.043060

Pairwise comparisons:

(Observed p-value below diagonal, permuted p-value above diagonal)

1 2 3 5 7 9

1 0.5380000 0.6650000 0.8540000 0.0590000 0.046

2 0.5282076 0.8820000 0.3270000 0.6480000 0.559

3 0.6468981 0.8809688 0.4620000 0.4420000 0.379

5 0.8500822 0.3317161 0.4825647 0.0200000 0.011

7 0.0566343 0.6407747 0.4688704 0.0165584 0.794

9 0.0434862 0.5295390 0.3830110 0.0091688 0.7722660

**> dist.t3 # 16S soil**

Permutation test for homogeneity of multivariate dispersions

Permutation: free

Number of permutations: 999

Response: Distances

Df Sum Sq Mean Sq F N.Perm Pr(>F)

Groups 7 0.05119 0.0073129 4.7308 999 0.001 ***

Residuals 236 0.36481 0.0015458

---

Signif. codes: 0 ‘***’ 0.001 ‘**’ 0.01 ‘*’ 0.05 ‘.’ 0.1 ‘ ’ 1

Pairwise comparisons:

(Observed p-value below diagonal, permuted p-value above diagonal)

1 2 3 5 7 9 11 13

1 8.7700e-01 5.0000e-02 1.0000e-03 6.0000e-02 1.0000e-02 1.0000e-03 0.007

2 8.7035e-01 6.3000e-02 1.0000e-03 8.9000e-02 1.9000e-02 1.0000e-03 0.024

3 3.8715e-02 7.4086e-02 4.0000e-02 7.1400e-01 8.4500e-01 1.9100e-01 0.564

5 5.3112e-06 4.0466e-05 3.7731e-02 6.0000e-03 3.2000e-02 3.2300e-01 0.161

7 5.2127e-02 1.0219e-01 7.0321e-01 6.1211e-03 4.6700e-01 4.6000e-02 0.291

9 6.8351e-03 1.9589e-02 8.2379e-01 3.1001e-02 4.8615e-01 2.0600e-01 0.655

11 7.7709e-05 4.6176e-04 1.8310e-01 3.2948e-01 5.1248e-02 1.9689e-01 0.521

13 9.5279e-03 2.1375e-02 5.6273e-01 1.6736e-01 3.1211e-01 6.5370e-01 5.4121e-01

**> dist.t4 # ITS soil**

Permutation test for homogeneity of multivariate dispersions

Permutation: free

Number of permutations: 999

Response: Distances

Df Sum Sq Mean Sq F N.Perm Pr(>F)

Groups 5 0.25738 0.051476 2.9098 999 0.015 *

Residuals 139 2.45898 0.017691

---

Signif. codes: 0 ‘***’ 0.001 ‘**’ 0.01 ‘*’ 0.05 ‘.’ 0.1 ‘ ’ 1

Pairwise comparisons:

(Observed p-value below diagonal, permuted p-value above diagonal)

1 2 3 5 7 9

1 0.01700000 0.23900000 0.85600000 0.05900000 0.125

2 0.01559845 0.02700000 0.00100000 0.53400000 0.634

3 0.26680358 0.02495234 0.14400000 0.15800000 0.267

5 0.84991942 0.00083406 0.15323977 0.01100000 0.029

7 0.05433724 0.54623469 0.16086438 0.01024285 0.981

9 0.10751451 0.60413746 0.26867577 0.03429609 0.98502734
